# cuSCNN: A Secure and Batch-Processing Framework for Privacy-Preserving Convolutional Neural Network Prediction on GPU

**DOI:** 10.3389/fncom.2021.799977

**Published:** 2021-12-23

**Authors:** Yanan Bai, Quanliang Liu, Wenyuan Wu, Yong Feng

**Affiliations:** ^1^Chongqing Key Laboratory of Automated Reasoning and Cognition, Chongqing Institute of Green and Intelligent Technology, Chinese Academy of Sciences, Chongqing, China; ^2^University of Chinese Academy of Sciences, Beijing, China; ^3^Chongqing School, University of Chinese Academy of Sciences, Chongqing, China

**Keywords:** privacy-preserving, convolutional neural network, homomorphic encryption, GPU computation, deep learning, cloud computing

## Abstract

The emerging topic of privacy-preserving deep learning as a service has attracted increasing attention in recent years, which focuses on building an efficient and practical neural network prediction framework to secure client and model-holder data privately on the cloud. In such a task, the time cost of performing the secure linear layers is expensive, where matrix multiplication is the atomic operation. Most existing mix-based solutions heavily emphasized employing BGV-based homomorphic encryption schemes to secure the linear layer on the CPU platform. However, they suffer an efficiency and energy loss when dealing with a larger-scale dataset, due to the complicated encoded methods and intractable ciphertext operations. To address it, we propose cuSCNN, a secure and efficient framework to perform the privacy prediction task of a convolutional neural network (CNN), which can flexibly perform on the GPU platform. Its main idea is 2-fold: (1) To avoid the trivia and complicated homomorphic matrix computations brought by BGV-based solutions, it adopts GSW-based homomorphic matrix encryption to efficiently enable the linear layers of CNN, which is a naive method to secure matrix computation operations. (2) To improve the computation efficiency on GPU, a hybrid optimization approach based on CUDA (Compute Unified Device Architecture) has been proposed to improve the parallelism level and memory access speed when performing the matrix multiplication on GPU. Extensive experiments are conducted on industrial datasets and have shown the superior performance of the proposed cuSCNN framework in terms of runtime and power consumption compared to the other frameworks.

## 1. Introduction

**Figure 1:**
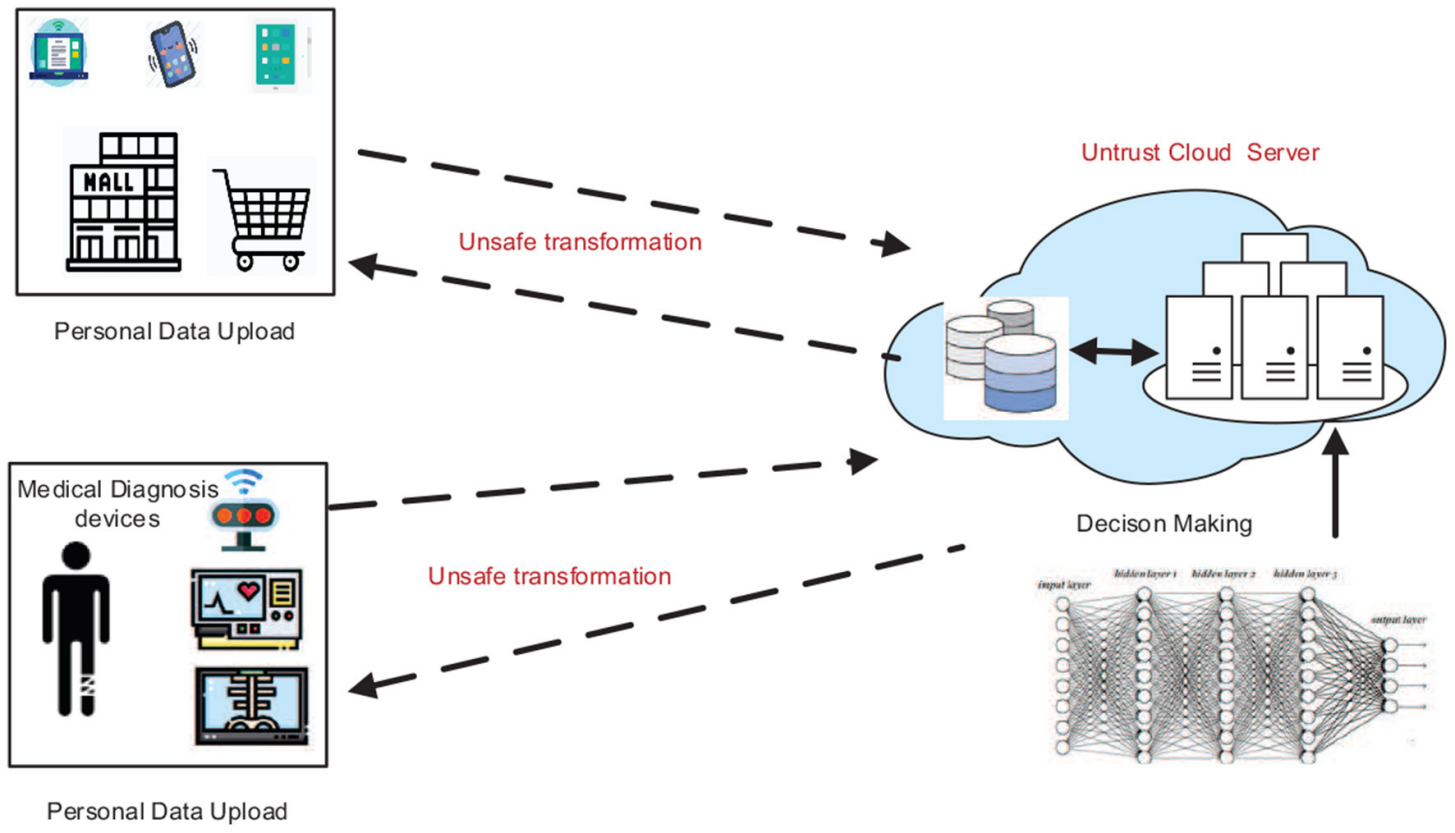
The privacy question of the deep learning model deployed on an untrusted cloud.

Deep learning (DL) has been applied to lots of fields [e.g., visual recognition (He et al., 2016), medical diagnosis (Shen et al., 2017), risk assessment (Deng et al., 2021a,b), and a recommender system (Shi et al., 2020; Wu et al., 2021a,b)], which achieves a superior performance in comparison with human cognition. The DL with a complex neural network (DNN) structure usually requires massive data for training a high-accuracy model. To alleviate the cost of using DL models, cloud providers (e.g., Amazon, Alibaba, Microsoft) are now providing Deep Learning as a Service (DLaS) that offers DL model training and inference APIs for clients. For example, Google AI^1^ provides a series of APIs for AI services (e.g., image classification, personalization recommendation, etc.). By calling these APIs, the client can upload their plaintext data to the cloud, then receive the analysis results (e.g., predication or classification task) by paying certain fees, as shown in Figure 1. Due to the fact that users' queries often involve personal privacy information, such as X-ray images or user's behavior trajectory data (Wu et al., 2020), a natural yet essential question about the protection of privacy has been raised: *if massive personal data are collected for model training and prediction, will the disclosing of user-sensitive information increase?* (Riazi et al., 2019; Liu et al., 2020).

Although those cloud providers claim that they will never leak or use users' data for commercial purposes, the increasing number of user data leaks tell us that there is no guarantee on what they promised (Abadi et al., 2016). An intuitive solution to protect user's privacy during DL inference is to give users propriety to download the model from the server and run the model on their platform locally. Nevertheless, this is an undesirable result for the model-holder (e.g., company or hospital) for at least two reasons: (1) The well-trained DL model is considered as the core intellectual property for companies, which is built on the massive collection of data. To avoid the loss of profits, companies require confidentiality to preserve their competitive advantage. (2) The well-trained DL model is known to reveal information about the underlying data used for training. In the case of medical data, this reveals sensitive information about other patients, violating their privacy and perhaps even HIPAA regulations (Assistance, 2003).

Therefore, the target of our work is to design a privacy-preserving service framework where both the model-holder and client can use the well-trained DL model and private data without worries. Two important requirements should be considered:

1. For protecting the privacy of the data owner, their sensitive queries should not be revealed to the model-holder;
2. For the proprietary of the model-holder, the DL model should not be revealed to users, in order to preserve their competitive advantage.

Following this mainstream, several solutions based on various secure computing technologies have been proposed, such as homomorphic encryption (HE)-based (Dowlin et al., 2016), multi-party computing (MPC)-based (Rouhani et al., 2018), and mixed-based solutions (Juvekar et al., 2018). Among them, HE (Gentry and Craig, 2009) is an intuitive yet promising way to evaluate it, which considers the whole neural network as a function and evaluates it in the ciphertext domain thoroughly, such as CryptoNet (Dowlin et al., 2016). Secure multi-party computing is another option for secure function evaluation. Secret sharing (SS) (Shamir, 1979) and garbled circuits (GC) (Yao, 1986) are two representational methods. They can transform a neural network model into an oblivious form and evaluate it with secure two-party computation, such as MinONN (Liu et al., 2017). Besides, mixed-based solutions have been proposed to obtain better performance with trade-off for each advantage, such as Gazzle (Juvekar et al., 2018).

We notice that the CNN inference task requires a lot of inner product operations to finish the convolutional layer. The existing mix-based methods usually adopt the Chinese Remainder Theorem (CRT)-based Single Instruction Multiple Data (SIMD) schemes to execute inner product operations of privacy-preserving CNN. However, it is time-consuming, since rotating operations in privacy-preserving CNN are required to sum up the results among slots. Different with the above solutions, we adopt the GSW-based method to design the matrix multiplication method in the ciphertext space, which is the main motivation of this study. The advantage of the GSW-based solution is that the ciphertext operation is a natural matrix operation without the expensive rotate-and-add strategy. Furthermore, with the rapid development of graphics processing hardware, a GPU is becoming the standard for cloud providers, where CUDA programming makes it possible to harness the computation power of GPU efficiently. Therefore, the use of GPU technology to accelerate matrix multiplication is another important motivation of this study.

On this basis, we introduce cuSCNN, a practical realization of a mixed-based framework that supports the privacy-preserving prediction of convolutional neural networks (CNNs). CNN is one of the most popular neural network architectures in DL. Generally, a CNN model consists of convolutional layers, activation, pooling, and fully connected layers. Convolutional and fully connected layers have linear properties, while activation and pooling are non-linear layers. For cuSCNN, it employs HE to perform the linear operations (e.g., homomorphic addition and multiplication) in each layer, while conducting the non-linear activation functions and pooling operations collaboratively by employing HE and GC jointly. The main contribution of this paper is as follows:

- We propose cuSCNN, an efficient and privacy-preserving neural network prediction framework that keeps user and server data secure. We employ the optimized homomorphic matrix computations for the linear operations in CNN, while adopting GC technology to execute the non-linear operations. Our secure matrix-based computation implements linear operations in the batch mode when dealing with a large-scale dataset.
- We introduce an efficient and natural GSW-based homomorphic matrix encryption scheme to support secure matrix multiplication and addition operations. Furthermore, we propose a hybrid optimization approach to matrix multiplication on GPU to improve the computation efficiency, which combines dual-optimization for I/O and computation.
- We implement cuSCNN on real-world data with varied CNN models and evaluate its performance on the industrial dataset. The experimental results show the superiority and effectiveness of cuSCNN in terms of runtime and power consumption, compared with state-of-the-art works.

The rest of this paper is organized as follows. Section 2 gives the preliminaries. Section 3 overviews the cuSCNN framework. Section 4 gives the implementation details of the cuSCNN framework. Section 5 evaluates the performance of cuSCNN. Finally, section 6 concludes this paper.

## 2. Preliminaries

### 2.1. Related Work

#### 2.1.1. Privacy-Preserving Neural Network Inference Framework

As the representative solution of homomorphic encryption-based solutions, CryptoNets (Dowlin et al., 2016) can evaluate the trained neural network in the ciphertext domain via utilizing leveled homomorphic encryption (LHE). However, the most critical limitation of CryptoNets is that the computational complexity drastically increases as the depth of layers in the NN model increases. Moreover, due to only adopting the LHE, non-linear functionalities such as the ReLU activation function in CryptoNets cannot be supported. To support the non-linear functionalities and pooling operations, DeepSecure (Rouhani et al., 2018) leverages GC as its backbone cryptographic engine. It can support various activations in the DL model. However, since multiplication is an atomic operation in the DL model and the number of Boolean gates in the multiplication circuit grows 2*x* times concerning the bit width of operands, together with multiple interactions between participants, DeepSecure requires an extensive communication overhead when performing secure privacy-preserving prediction. MiniONN (Liu et al., 2017) transforms a neural network model into an oblivious form and evaluates it with secure two-party computation. In detail, it utilizes the GC to compute the non-linear activation function while incorporating SS and HE-based methods to run the linear operations in the DNN model. Moreover, GAZELLE (Juvekar et al., 2018) is another mixed-protocol solution that uses an intricate combination of HE and GC to carry out the inference phase of the DNN model, which utilizes the GC to perform the non-linear activation function and uses lattice-based HE with packing technology to execute linear operations. As a result, GAZELLE improves the runtime of private inference and reduces communication between the user and the cloud. To improve the efficiency of the ciphertext computations, FALCON (Li et al., 2020) exploits the Fast Fourier transform to accelerate the homomorphic computations in the convolutional and fully connected layers. Unlike the method mentioned above, we introduce GSW-based secure matrix computations to implement the linear layers and leverage the GPU to accelerate the computation efficiency of the proposed approach.

#### 2.1.2. Matrix-Based Homomorphic Encryption Scheme

Matrix-based computations are the core yet time-consuming operations in the neural network. In this context, some matrix-based homomorphic encryption schemes have been proposed. Based on the SIMD technology, Wu and Haven et al. proposed a safety inner product method on packed ciphertexts (Wu and Haven, 2012). Lu et al. (2016) modified the matrix-vector multiplication for secure statistical analysis over HElib. Duong et al. (2016) proposed a homomorphic matrix multiplication scheme on the packed ciphertext over RLWE. Later, Mishra et al. (2017) designed an enhanced version of the matrix multiplication, but there were useless terms in the ciphertexts. Besides, it is only suitable for a one-depth homomorphic multiplication scenario, due to the significant expansion rate of ciphertexts. Wang et al. (2017) modified Duong's methods for flexible matrix computation, but their modification was much less efficient for matrices of larger size. Jiang et al. (2018) presented a novel matrix encoding method that can encrypt more than one matrix in a single ciphertext and adapted an efficient evaluation strategy for generic matrix operations via linear transformations. However, the methods mentioned above were all constructed based on the second-generation HE scheme with unnecessary key switching, which suffers efficiency and precision loss when dealing with large-scale data. Hiromasa et al. (2016) first conducted a GSW-FHE scheme for matrix homomorphism computations (i.e., HAO). They optimized the bootstrapping technique proposed by Alperin-Sheriff and Peikert (2014). However, all these improvements target binary plaintext, which dramatically restricts its application in the real world.

### 2.2. Notations and Definitions

Assume that vectors are in column form and are written using bold lower-case letters e.g., **x**, while bold capital letters are used to denote matrices, e.g., **X**. We introduce gadget matrix **G** and the function *G*^−1^ by lemma 1. In order to facilitate readers to understand, the meanings of the notations mentioned in the encryption scheme are shown in Table 1.

**Table 1:** Meaning of notation in the homomorphic encryption scheme.

**Notations**	**The meaning**
∥**x**∥_∞_	The maximum norm of **x**
∥**x**∥_2_	The Euclidean norm of **x**
<**x**, **y**>	The inner product of two vectors **x** and **y**
*x*_*i*_	The *i*th element of vector **x**
$\left[\text{X}||\text{Y}\right]\in {\mathbb{Z}}^{m\times \left(n_{1}+n_{2}\right)}$	The column concatenation of **X** with **Y**, where $\text{X}\in {\mathbb{Z}}^{m\times n_{1}},\text{Y}\in {\mathbb{Z}}^{m\times n_{2}}$
$\left[\frac{\text{Y}}{\text{X}}\right]\in {\mathbb{Z}}^{\left(m_{1}+m_{2}\right)\times n}$	The row concatenation of $\text{X}\in {\mathbb{Z}}^{m_{1}\times n}$ with $\text{Y}\in {\mathbb{Z}}^{m_{2}\times n}$
**X**_*i*_	The *i*th column vector of **X**
**X**(*p*:*q, r*:*s*)	The submatrix consisting of rows *p* to *q* and columns *r* to *s* of the matrix **X**.
$a\overset{U}{\leftarrow }\text{D}$	*a* is chosen from set **D** uniformly at random
**I**_*r*_	The identity matrix with size of *r* × *r*
${\text{X}}_{ij}\in {\left\{0,1\right\}}^{r\times r}$	The matrix with 1 in the position (*i*,*j*) and 0 in the others
λ	Security parameters, the scheme can resist 2^λ^ attacks
*modq*	Modulus *q* with the range of values is [−(*q* − 1)/2, (*q* − 1)/2]
*round*(*x*)	Rounding *x* ∈ ℝ
⌈*x*⌉	Rounding up *x* ∈ ℝ
⌊*x*⌋	Rounding down *x* ∈ ℝ

**Lemma 1** **(Micciancio and Peikert, 2012)**. *Let matrix* $\text{C}\in {\mathbb{Z}}_{q}^{n\times m}$, *there are a fixed and primitive matrix* $\text{G}\in {\mathbb{Z}}_{q}^{n\times nl}$ *and a deterministic, randomized function* *G*^−1^ *that can be calculated by*: ${\mathbb{Z}}_{q}^{n\times m}\to {\mathbb{Z}}_{q}^{nl\times m}$ *such that* $\text{X}\overset{R}{\leftarrow }G^{-1}\left(\text{C}\right)$ *is sub-Gaussian with parameter O(1) and always satisfies* **GX** = **C**.

Let *l* = ⌊log*q*⌋ + 1 and **g**^*T*^ = (2^0^, 2^1^, …, 2^*l*−1^), **I**_*n*_ is the unit matrix with *n* rank, then the gadget matrix can be defined as $\text{G}:={\text{I}}_{n}⊗{\text{g}}^{T}\in {\mathbb{Z}}_{q}^{n\times nl}$.

### 2.3. GSW-Based Homomorphic Matrix Encryption Scheme

Generally, a HE scheme consists of four algorithms HE=(Keygen, Enc, Dec, Eval) and can be illustrated as follows:

- *KeyGen*(*params*): Given the security parameter λ, the main function of *KeyGen*(*params*) is to produce a secret key **sk**, a public key **pk**, and a public evaluation key **evk**.
- *Enc*_**pk**_(*m*): Based on the created public key **pk**, the encryption algorithm encrypts a plaintext *m* ∈ **M** into a ciphertext *c* ∈ **C**.
- *Dec*_**sk**(*c*)_: Using the created secret key **sk**, it can recover the original plaintext *m* from the ciphertext *c*.
- *Eval*_*evk*_(*f, c*_1_, …, *c*_*l*_): Under the ciphertext space C with the evaluation key **evk**, the ciphertext *c*_*f*_ can be calculated by using the function $f:M^{l}\to M$ to *c*_1_, …, *c*_*l*_

The original GSW scheme is proposed by Gentry, Sahai, and Waters (Gentry and Craig, 2009). It adopts the approximate eigenvector method based on the plaintext space **M** to construct the ciphertext space **C**. Based on this scheme, Bai et al. proposed a homomorphic matrix encryption scheme (Bai et al., 2020), which can be described as follows:

Given the security parameter λ and the multiplication depth of circuit *L*, *l* = ⌊log*q*⌋ + 1. The integer modulus is *q* = *q*(λ, *L*): = 2^*l*−1^, the lattice dimension *n* = *n*(λ, *L*), and the noise distribution **χ** = **χ**(λ, *L*) follows a sub-Gaussian distribution over ℤ. Meanwhile, let *m* = *m*(λ, *L*): = *O*((*n* + *r*)*l*), and *N*: = (*n* + *r*)*l*. $\text{G}={\text{I}}_{n+r}⊗{\text{g}}^{T}\in {\mathbb{Z}}_{q}^{\left(n+r\right)\times N}$ can be calculated, where **g**^*T*^ = {2^0^, 2^1^, …, 2^*l*−1^}.

- HE.KeyGen(*n, q*, **χ**, *m*): The key generation method mainly includes two parts, i.e., the secret key **sk** and public key **pk**:
  - For **sk**, it first samples a secret key matrix $\bar{\text{S}}\leftarrow \chi^{r\times n}$, then the secret key matrix can be obtained as follows:
    (1) $$\begin{array}{r} \text{S}:=\left[{\text{I}}_{r}||-\bar{\text{S}}\;\right]\in {\mathbb{Z}}_{q}^{r\times \left(n+r\right)} \end{array}$$
  - For **pk**, it first generates a uniformly random matrix $\text{A}\overset{U}{\leftarrow }{\mathbb{Z}}_{q}^{n\times m}$, noise matrix $\text{E}\overset{R}{\leftarrow }\chi^{r\times m}$, and $R_{ij}\overset{U}{\leftarrow }{\left\{0,1\right\}}^{m\times N}$ (for all *i, j* = 1, …, *r*), then the public key matrix **B** is:
    (2) $$\begin{array}{r} \text{B}:=\left[\frac{\bar{\text{S}}\text{A}+\text{E}}{\text{A}}\right]\in {\mathbb{Z}}_{q}^{\left(n+r\right)\times m} \end{array}$$
    (3) $$\begin{array}{r} {\text{P}}_{ij}:=\text{B}{\text{R}}_{ij}+\left[\frac{{\text{M}}_{ij}\text{S}}{0}\right]\text{G}\in {\mathbb{Z}}_{q}^{\left(n+r\right)\times N} \end{array}$$
  Hence, the output of keygen(*n, q*, **χ**, *m*) is **sk**: = **S**, **pk**: = {**P**_*i,j*_, **B**||1 ≤ *i, j* ≤ *r*}.
- HE.SecEnc(**sk**, **M**): Sample the random matrix $\bar{\text{A}}\overset{U}{\leftarrow }{\mathbb{Z}}_{q}^{n\times N}$ and $\text{E}\overset{R}{\leftarrow }\chi^{r\times N}$, then the ciphertext **C** can be computed by :
  (4) $$\begin{array}{r} \text{C}:=\left[\frac{\bar{\text{S}}\bar{\text{A}}+\text{E}}{\bar{\text{A}}}\right]+\left[\frac{\text{M}\text{S}}{0}\right]\text{ G}\in {\mathbb{Z}}_{q}^{\left(n+r\right)\times N} \end{array}$$
- HE.PubEnc(**pk**,**M**): Sample a random matrix $R\overset{U}{\leftarrow }{\left\{0,1\right\}}^{m\times N}$, and the ciphertext can be denoted by
  (5) $$\begin{array}{r} \text{C}:=\text{B}\text{R}+\overset{r-1}{\underset{i=0}{\sum }}\overset{r-1}{\underset{j=0}{\sum }}M_{i,j}\cdot {\text{P}}_{i,j}\in {\mathbb{Z}}_{q}^{\left(n+r\right)\times N} \end{array}$$
- HE.Dec(**S**,**C**): The processing of the decryption algorithm can be described as follows:
  Step 1: Compute the matrix $\text{H}=\text{S}\text{C}\in {\mathbb{Z}}_{q}^{r\times N}$;
  Step 2: Denote the matrix ${\text{H}}_{i,j}^{\prime }\in {\mathbb{Z}}_{q}^{r\times rl}$, where *i* ∈ {1, 2, …, *r*}, and *j* ∈ {1, 2, …, *rl*}. Meanwhile, the noise matrix **E**′ has the same size as **H**′. Hence,
    (6) $$\begin{array}{r} {\text{H}}^{\prime }={\text{E}}^{\prime }+\left[\begin{array}{cccccc} {\text{M}}_{0,0} & \cdots & 2^{l}{\text{M}}_{0,0}\cdots & {\text{M}}_{0,r} & \cdots & 2^{l}{\text{M}}_{0,r}\\ \vdots & \vdots & \vdots & \vdots & \vdots & \vdots \\ {\text{M}}_{r,0} & \cdots & 2^{l}{\text{M}}_{r,0}\cdots & {\text{M}}_{0,r} & \cdots & 2^{l}{\text{M}}_{0,r} \end{array}\right] \end{array}$$
  Step 3: Recover each element (i.e., *m*_*i,j*_) in the plaintext matrix **M** via the function Dec1Num(**H**′(*i*, *jl* :(*j* + 1)*l* − 2)), where 1 ≤ *i* ≤ *r* and 1 ≤ *j* ≤ *r*. The implementation details of Dec1Num can be found in Bai et al. (2020).
- HE.MatAdd(*C*_1_, *C*_2_): Given the two ciphertext matrices ${\text{C}}_{1}\in {Ƶ}_{q}^{\left(n+r\right)\times N}$ and ${\text{C}}_{2}\in {Ƶ}_{q}^{\left(n+r\right)\times N}$, the homomorphic matrix addition can be defined as:
  (7) $$\begin{array}{r} {\text{C}}_{add}={\text{C}}_{1}+{\text{C}}_{2}\in {\mathbb{Z}}_{q}^{\left(n+r\right)\times N} \end{array}$$
- HE.MatMult(**C**_1_, **C**_2_): For **C**_1_, ${\text{C}}_{2}\in {\mathbb{Z}}_{q}^{\left(n+r\right)\times N}$, it first computes $G^{-1}\left({\text{C}}_{2}\right)\in {0,1}^{N\times N}$, then outputs:
  (8) $$\begin{array}{r} C_{mult}:=C_{1}\;\cdot \;C_{2}=C_{1}G^{-1}(C_{2})\in {\mathbb{Z}}_{q}^{(n+r)\times N} \end{array}$$

To implement the privacy-preserving linear operations in cuSCNN, two kinds of homomorphic computation should be supported: HE.MatAdd and HE.MatMul. HE.Mat means that we can encrypt the plaintext matrix as approximate eigenvalues of the ciphertext matrix correspondingly, where the secret key is the eigenvector. Since the ciphertext calculation of GSW is based on the matrix computation, which cannot cause the expansion of the ciphertext dimension, it can significantly eliminate the unnecessary key conversion brought by BGV-based solutions. HE.MatAdd represents the homomorphic addition between two matrixes in the ciphertext domain, while HE.MatMul means the homomorphic multiplication between two matrices.

### 2.4. GPU-Based Computing

**Figure 2:**
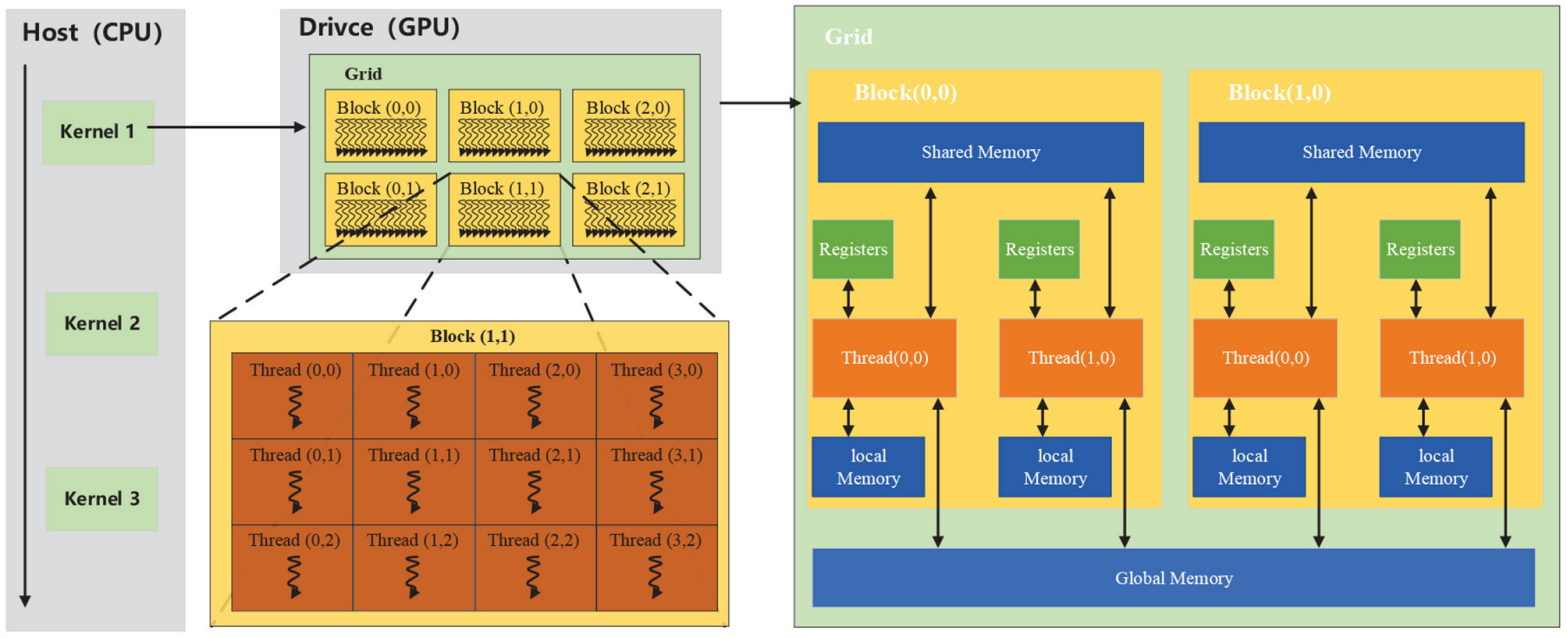
CUDA kernel and memory hierarchies.

A graphics processing unit (GPU) is a specialized electronic circuit designed to rapidly manipulate and alter memory to accelerate the creation of images in a frame buffer intended for output to a display device. GPU adopts a large number of computing units and ultra-long pipelines, but it only has straightforward control logic and eliminates cache. Their highly parallel structure makes them more efficient than CPUs for algorithms that process large data blocks in parallel. CUDA (an acronym for Compute Unified Device Architecture) is a parallel computing platform and application programming interface (API) model created by Nvidia, which allows GPU to be compatible with various programming languages (e.g., C++, Fortan, and Python) and applications. The CUDA platform is a software layer that gives direct access to the GPU's virtual instruction set and parallel computational elements to execute compute kernels. In CUDA, *kernels* are functions that are executed on GPU, which are executed by a batch of threads. Meanwhile, the batch of threads is organized as a grid of thread blocks. Thus, a GPU with more blocks can execute a CUDA program in less time than a GPU with fewer blocks. As shown in Figure 2, threads in a block are organized into small groups of 32 called *wraps* for execution on the processors, and *wraps* are implicitly synchronous; however, threads in different blocks are asynchronous. CUDA assumes that the CUDA kernel, i.e., CUDA program, is executed on a GPU (drive), and the rest of the C program is executed on the CPU (host). CUDA threads access data from multiple memory hierarchies. Each thread has a private register and local memory, and each thread block has shared memory visible to all threads within the same thread block. All threads can access global memory.

## 3. The cuSCNN Framework

In this section, we design a privacy-preserving CNN prediction framework. Consider a cloud-based medical diagnosis scenario where a user wants to know his health status from an X-ray image. In our setting, we have two roles:

1. The cloud service provider (*S*) holds trained classifier models and has resources for storage and processing. He has a business interest in computation and making predictions on encrypted data from clients.
2. Clients (*C*) are the customers of the service provider. He uploads his private image to the cloud server via API interference and receives the result by paying specific service fees.

### 3.1. Overview

We introduce the execution flow of cuSCNN at a high level. Suppose that client *C* owns the input data (e.g., an X-ray image) and the server *S* holds a convolutional neural network model. For client *C*, the input is private. For server *S*, the details of the trained CNN model are also private, which includes the weights of convolutional and fully connected layers. The target of cuSCNN is to preserve privacy for the client and server when performing the CNN model.

**Hypothesis**: Both *S* and *C* are *semi-honest* Paverd et al. (2014), we presume they follow the cuSCNN protocol and never deviate from it, although they might attempt to infer more information based on the data they receive and transmit. Specifically, *C* leaks no information about the input contents, intermediate calculation result, and classified results to the cloud. The input data are factual, never using fake data. For the cloud server, the weights of the CNN model are kept secret from the client, but it does not hide the model architecture.

**Figure 3:**
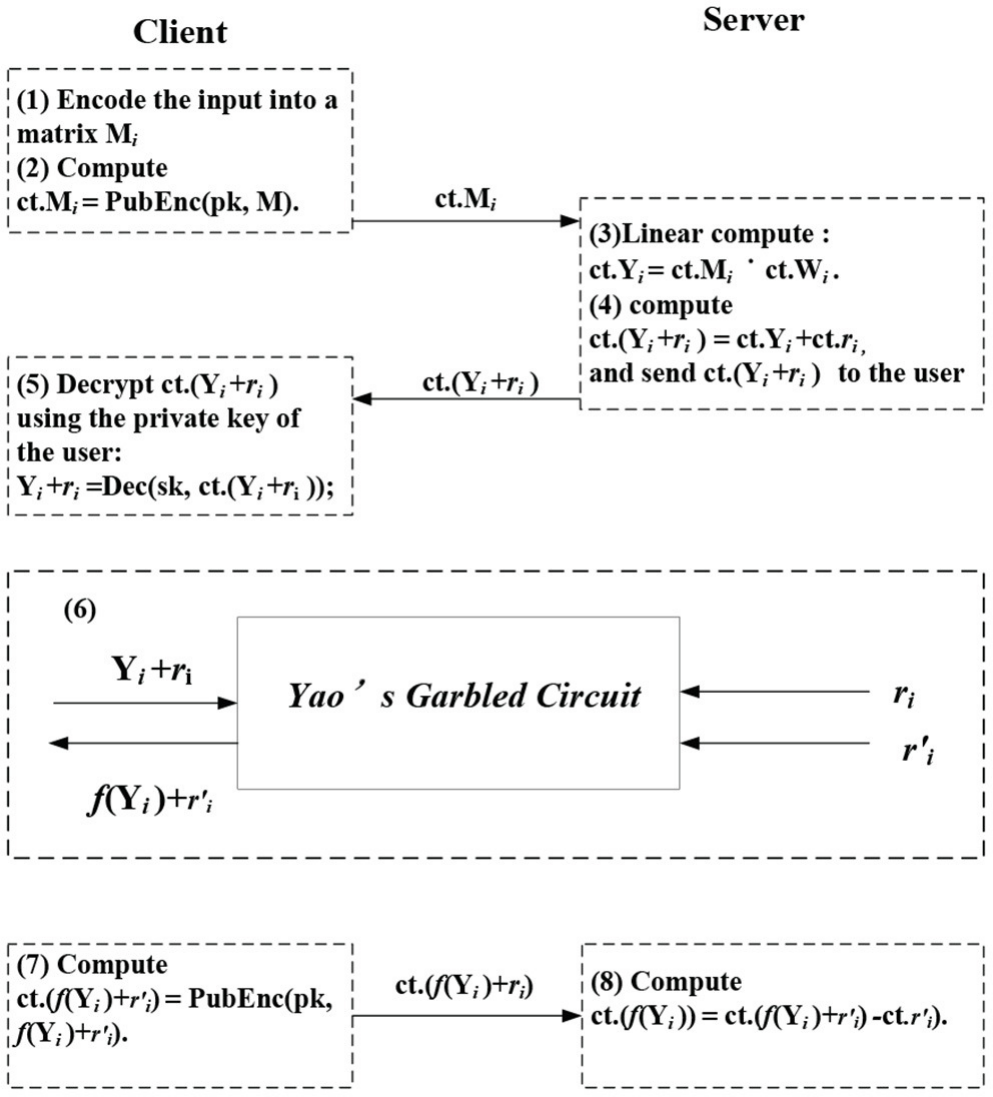
Security interactive computation protocol of cuSCNN.

There are two phases of the framework, including off-line and online phases. In the off-line phase, the cloud generates shares **r** and **r′** used for Yao's garbled circuit. Besides, the cloud encrypts these shares and their weight matrices using the client's public key. In the online phase, the convolutional and fully connected operations are linear computations, while the activation functions are non-linear. The execution flow of the proposed cuSCNN is indicated in Figure 3.

- **Evaluate linear layers (i.e., Conv and FC layer)**: For *i* ∈ [1, 2, …*l*], *l* is the number of hidden layers, *C* firstly encodes the input data into matrix **M**_*i*_, then it encrypts **M**_*i*_ via calling the public-key encryption algorithm, denoted by *ct*.**M**_*i*_ = PubEnc(pk, **M**_*i*_), and uploads *ct*.**M**_*i*_ to the cloud server *S*. *S* utilizes the encryption scheme to execute matrix-matrix multiplication in convolutional layers and vector-matrix multiplication fully connected layers.
  Take the Conv layer, for instance, *C* feeds the convolutional layer with an encrypted input matrix *ct*.**M**_*i*_, *S* computes **Y**_*i*_ = **W**_*i*_·*ct*.**M**_*i*_. **W** is the filter's matrices. The fully connected layer is similar except for homomorphic vector-matrix multiplication.
- **Evaluate non-linear functions (i.e., activation and pooling layer):** *S* and *C* perform designed secure computation protocols, i.e., Yao's garbled circuit to keep data secure. Concretely, for layer *i*, *S* homomorphically adds encryption of the share **r**_*i*_ to obtain the encryption of **Y**_*i*_ + **r**_*i*_, and send it to the client. The client decrypts it using his private key to obtain the plaintext **Y**_*i*_ + **r**_*i*_. Next, **Y**_*i*_ + **r**_*i*_ is held by the client and **r**_*i*_ and **r**′_*i*_ are held by the server as inputs are conducted in garbled circuit evaluation. The output of it is **r**′_*i*_ (the activation function is denoted by *f*). Then the client encrypts it using their public key and transmits the ciphertext to the server, the server homomorphically adds the encryption of the share **r**′_*i*_ to get the encryption of *f*(**Y**)_*i*_.

### 3.2. Neural Networks Architecture

In DL, CNN is a popular category of neural network, most commonly applied to analyzing visual imagery. It usually consists of an input and output layer, as well as multiple hidden layers. In most cases, a CNN takes an input and processes it through a sequence of hidden layers to classify it into one of the potential classes. Hidden layers typically consist of a series of linear (e.g., convolutional, fully connected) layers and non-linear (e.g., activation function and pooling) layers.

For the Conv layer, the convolution operator forms the fundamental basis of the convolutional layer. It has convolutional kernels with size *k* × *k*, a stride of (*s, s*), and a mapcount of *h*. Given an image *I* ∈ ℝ^*w*×*w*^ and a convolution kernel **W** ∈ ℝ^*k*×*k*^, the convolved image $\text{Y}\in {\mathbb{R}}^{d_{k}\times d_{k}}$ can be computed as follows:


(9) $$\begin{array}{r} \text{Y}=Conv{\left(I,\text{W}\right)}_{i^{\prime },j^{\prime }}=\underset{0\leq i,j\leq k}{\sum }W_{i,j}\cdot I_{s\cdot i^{\prime }+i,s\cdot j^{\prime }+j} \end{array}$$

where the range of (*i*′,*j*′) is $\left[0,\left\lceil \frac{\left(w-k\right)}{s}\right\rceil +1\right]$, and ⌈·⌉ denotes the least integer greater than or equal to the input. For multiple kernel cases, it can be expressed as:


(10) $$\begin{array}{l} Y=Conv(I,W)=\left(\left(Conv(I,W^{\left(0\right)}\right),\cdots ,Conv\left(I,W^{\left(h-1\right)}\right)\right)\\ \;\in {\mathbb{R}}^{d_{k}\times d_{k}\times h} \end{array}$$

For the FC layers, it connects *n*_*I*_ nodes to *n*_*O*_ nodes, which can form as the matrix-vector multiplication of an *n*_*O*_ × *n*_*I*_ matrix. Note that the output of the convolutional layer has a form of tensor, so it should be flattened before the FC layer.

## 4. cuSCNN Design

We next utilize a commonly used CNN in privacy protection work (Dowlin et al., 2016; Rouhani et al., 2018) to describe the design details. The network topology contains one convolutional layer, one fully connected layer with ReLU activation function, and the second fully connected layer applying the softmax activation function for probabilistic classification. Table 2 describes our neural networks to the MNIST dataset and summarizes the parameters.

**Table 2:** Layers description of CNN.

**Layers**	**Description**
Layer-1[Conv-1]	Input image: 28 × 28, kernel size: 5 × 5, stride: (1,1), number of output channels: 5, padding = VALID, activation = ReLU.
Layer-2[FC-1]	Fully connecting with 5 × 3 × 3 = 845 inputs and 100 outputs, activation = ReLU.
Layer-3[FC-2]	Fully connecting with 100 inputs and 10 outputs activation = softmax.

### 4.1. Encryption of Images

We assume that a neural network is trained with the plaintext dataset in the clear. For the CNN architecture in Table 2, *w* = 28, *k* = 5, *d*_*k*_ = 13, *s* = 2, and *h* = 5. Suppose that the client has a two-dimensional image *I* ∈ ℤ^*w*×*w*^. For 0 ≤ *i, j* < 5, 0 ≤ *i*′, *j*′ < 13, by taking the elements $I_{s\cdot i^{\prime }+i,s\cdot j^{\prime }+j}$, we extract the image feature to an extended matrix **M** with the size of 25 × 169. For bias, we add the vector [1, …, 1]^169^ to the first row. For a matrix **M** with the size of 26 × 169, it is blocked into *b*_*num*_ = 7 sub-matrices **M_b_** for parallel computation, where $b_{num}=\left\lceil N_{i}/\left(k^{2}+1\right)\right\rceil ,N_{i}=d_{k}\times d_{k}$. Since this CNN can deal with 846 images in FC-1, we design the framework to compute 846 images at once to achieve this maximum throughput. At the encryption phase, the client *C* encrypts the **M**_*b*_ using the public key of a HE scheme.

For PubEnc(*pk*, **M**_*b*_), we first sample a random matrix **R** ← {0, 1}^*m*×*N*^ uniformly, then the encrypted image can be computed by (11).


(11) $$\begin{array}{r} ct.{\text{M}}_{b}=PubEnc\left(pk,{\text{M}}_{b}\right):=\text{B}\text{R}+\underset{0\leq i,j\leq r}{\sum }M\left[i,j\right]\cdot {\text{P}}_{\left(i,j\right)}\in {\mathbb{Z}}_{q}, \end{array}$$

### 4.2. Encryption of Trained Model

The model provider encrypts the trained prediction model values such as multiple convolution kernel values **W** and weights (matrices) of FC layers.

The provider begins with a procedure for encrypting the multiple convolutional kernels. Each kernel is extended into a one-row vector of size *k*^2^, and the bias is connected to the first column. Hence, *h* kernels are expanded into a matrix with a size of 5 × 26. Then the provider pads (*k*^2^ + 1) − *h* (i.e., 21) rows with zeros to form a square matrix. Finally, the model provider encrypts the plaintext matrix into a ciphertext, denoted by *ct*.**W**_1_.

Next, the first FC and the second layer are specified by 100 × 846 and 10 × 101 matrices. They can pad 746 and 91 rows with zeros to become two square matrices. Then the model provider encrypts the two matrices respectively, and the ciphertexts are *ct*.**W**_2_ and *ct*.**W**_3_.

### 4.3. Homomorphic Evaluation of Neural Networks

The public cloud takes ciphertexts of the images from the data owner and the neural network prediction model from the model provider at the prediction phase. Since the data owner uses a batch of 864 different images, the FC-1 layer is specified as a matrix multiplication: ℤ^100×846^ × ℤ^846×846^ → ℤ^100×846^, and the FC-2 layer is represented as a matrix multiplication: ℤ^10×101^ × ℤ^101×101^ → ℤ^10×101^. The FC-1 layer inputs 846 computational image results to the FC-2 layer, and the FC-2 layer can deal with 101 images at once, so the FC-2 layer needs to execute nine times to finish the 846 image prediction task.

*Homomorphic Conv-1 layer*: For 0 ≤ *i* < 846, 0 ≤ *j* < 7, the public cloud takes the ciphertexts $ct.{\text{M}}_{b}^{\left(i,j\right)}$ and *ct*.**W**_1_, and it performs the following computation on‘ciphertexts:


(12) $$\begin{array}{r} ct.C_{1}\leftarrow \sum_{0\leq i<846,0\leq j<7}Mult(ct.M_{b}^{\left(i,j\right)},ct.W_{1})\in {\mathbb{Z}}_{q}^{(n+r)\times N}. \end{array}$$

*Secure activation layer*: In order to protect the convolutional result **Y** and safely compute the activation function, the framework adopts Yao's garbled circuits method similar to GAZALLE and FALCON. The ReLU function is defined by *f*(**x**) = *max*(**x**, 0), the cloud generates sharing *r* in the preprocessing phase, *C* and *S* share the input **x** additively, i.e., *S* holds *r*, while *C* holds *max*(**x**, 0)−*r*. The two parties jointly compute *GT* and *MUX* circuits to get *f*(**x**)+*r*′, which is sent to *C*. *C* loads the 846 images to form a square matrix **M**_2_, then we encrypt it into ciphertext *ct*.**M**_2_ and send it to the cloud.

*The FC-1 layer*: The cloud firstly performs a homomorphic addition operation to remove sharing, and then it carries out the homomorphic matrix multiplication:


(13) $$\begin{array}{r} ct.C_{2}\leftarrow Mult(ct.{M^{\prime }}_{2},ct.W_{2})\in {\mathbb{Z}}_{q}^{(n+r)\times N}. \end{array}$$

Next, the cloud and the user conduct the activation operation by the garbled circuit. Afterward, the user sends the ciphertext *ct*.**C**_3_ to the cloud.

*The FC-2 layer* The homomorphic evaluation in FC-2 is similar to FC-1, except for executing nine times to finish 846 image predictions.


(14) $$\begin{array}{r} ct.C_{3}^{\left(i\right)}\leftarrow Mult(ct.{M^{\prime }}_{3}^{\left(i\right)},ct.W_{3})\in {\mathbb{Z}}_{q}^{(n+r)\times N},0\leq i\;<9. \end{array}$$

The activation operation of FC-2 is a softmax function, since $y_{i}=\frac{e^{z_{i}+r^{\prime }}}{\overset{num\text{\_}out}{\underset{j=1}{\sum }}e^{z_{j}+r^{\prime }}}=\frac{e^{z_{i}}}{\overset{num\text{\_}out}{\underset{j=1}{\sum }}e^{z_{j}}}$, where *z*_*i*_ is the *i*th *i* ∈ [1, *num*_*out*] input elements of the last fully connected layer, *D* decrypts the ciphertext and gets the prediction result directly.

Please note that the plaintext of the scheme is a square matrix, and the length of the input vector is set to 846 (5 × 13 × 13 + 1) in the example. Thus, to maximize the use of plaintext space to improve operating efficiency, we need the number of input images to be 846. In the general case, the number of input images takes the max length of the fully connected layers input vectors in the proposed framework.

### 4.4. Hybrid Optimization Approach on GPU for Efficient Matrix-Based Computation

To improve homomorphic matrix multiplication efficiency and utilize the powerful computing ability of GPU, we propose a hybrid optimization approach to execute the homomorphic matrix multiplication on GPU.

Given two matrices **A** and **B** with the size of *r* × *r*, the straightforward way is to open a thread for computing each element of its output matrix **C**. For parallel matrix multiply operation, each thread loads a row of **A** (i.e., **A**(**i**, :)) and a column of **B** (**B**(:, **j**)), then **c**_**ij**_ can be computed via making an inner product of these two vectors (i.e., **c**_**ij**_ = **A**(**i**, :) · **B**(:, **j**)). However, the delay in accessing the shared memory on the GPU is quite significant (almost 100 clock cycles). For example, suppose that the matrix elements are stored in the memory following the rows first way, then a row of **A** can be saved in the memory continuously, and it can utilize the super large shared memory bandwidth of the GPU to load multiple elements with a short accessing delay. However, for the matrix **B** with a large size *r*, the memory address of elements in a column is internal with *r* elements. It means that most of the data are useless except the required column of elements in a load time. As a result, the memory access efficiency of this parallel method is appalling, since it is almost impossible for this access mode to hit the cache line.

**Figure 4:**
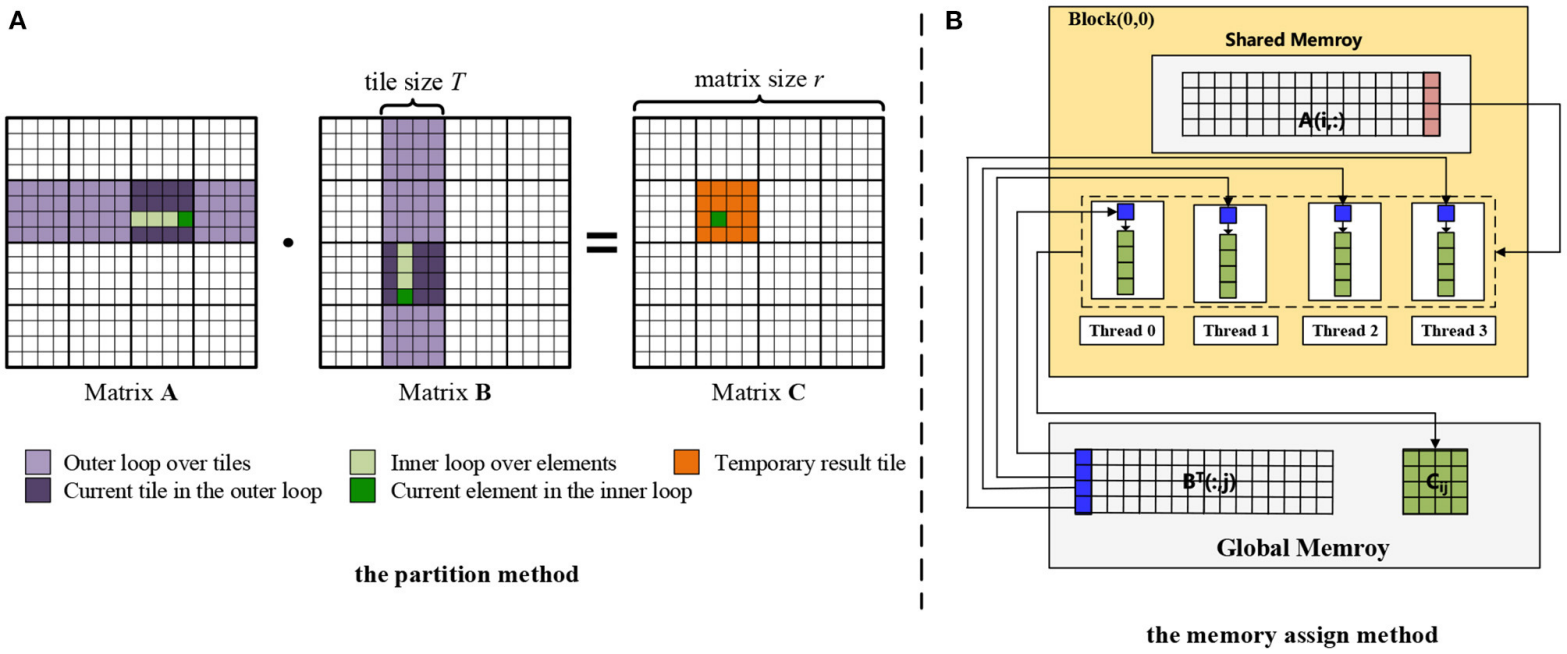
Hybrid optimization approach on GPU.

To address this problem, we introduce a partitioning algorithm for matrix multiplication computation on GPU. For the partition method as shown in Figure 4A, the key is to determine *how to maximize the use of limited shared memory space*. The shared memory (SM) is an on-chip cache located on the GPU, which can be as fast as the first level cache, and threads in the same thread block can exchange data through SM. The only disadvantage is that the capacity of SM is limited. To use this small piece of high-speed memory, we divide the matrix into a set of small pieces in each dimension. Suppose that the slice size is *T*, the output matrix **C**_00_ can be written as:


(15) $$\begin{array}{r} {\text{C}}_{00}=\overset{bk-1}{\underset{i=0}{\sum }}A_{0,i}\cdot B_{i,0} \end{array}$$

where $bk=\left\lceil \frac{r}{T}\right\rceil$ is the block numbers of matrices **A** and **B**. Note that the small slice matrix will degenerate into a single element when the small slice size *T* becomes 1. If the small piece is regarded as an element, the size of the whole matrix is reduced by *T* times.

Each piece of the output matrix **C** can assign a thread block with a group of threads to compute the result, where each thread corresponds to an element in the piece. In detail as shown in Figure 4B, each thread stores one element of block **B**(:, *j*) and one column of *C*_*ij*_ in its register. **A**(*i*, :) is stored in the shared memory of Block(0,0), which can be accessed by the threads in Block(0,0). Instead of using the inner product to perform matrix multiplication, we adopt the outer product to optimize the computation. For example, it first performs the outer product between the first column of **A**(:, 0) and the first row of **B**(0, :) and updates **C**_*ij*_. Then the **C**_*ij*_ is updated via **A**(:, 1) and **B**(1, :). Executing the iterations in a similar way until *T* times, the updated **C**_*ij*_ can be obtained. Finally, each thread stores one column of **C**_*ij*_ from its register to global memory.

As we know, the time complexity of naive matrix multiplication is *O*(*r*^3^). Due to leveraging the proposed partition method, the big matrices **A** and **B** can be divided into *bk* blocks with slice size *T*. For each slice, the time complexity is *O*(*T*) when calling *T* threads to perform it in parallel. Hence, the total time complexity of the proposed matrix multiplication on GPU is *O*((*bk*)^2^ × *T*).

### 4.5. Security Analysis

We prove that the encryption scheme defined above is IND-CPA secure under the LWE hardness assumption.

** Theorem 1**. *For any adversary* A *there exists an adversary such that* AdvCPA(A)<2AdvLWE(B).

*Proof*: ***G***_**0**_: A challenger C first initializes the encryption scheme and setup parameters, then generates a public key *pk* and a private key *sk*. The adversary A obtains the public key and selects two challenge plaintexts *m*_0_ and *m*_1_ from the plaintext space, and sends them to the challenger C. C chooses *b* ∈ [0, 1] at random, and encrypts *m*_*b*_ using the public key, then sends the ciphertext to adversary A. The adversary guesses the plaintext corresponding to the ciphertext and outputs *b*′. If *b* = *b*, the adversary attacks successfully, and the advantage of the adversary is $AdvCPA\left(A\right)=|Pr\left[b=b^{\prime }\;in\;G_{0}\right]-1/2|$.

***G***_**1**_: In ***G***_**1**_, the public key *pk*: = **P**_(*i, j*)_, **B** used in ***G***_**0**_ is substituted by a uniform random matrix $\text{B}\leftarrow {\mathbb{Z}}_{q}^{\left(n+r\right)\times m}$, and **P**_(*i, j*)_ is substituted by a uniform random matrix ${\text{P}}_{\left(i,j\right)}^{\prime }\leftarrow {\mathbb{Z}}_{q}^{\left(n+r\right)\times N}$. It is possible to verify that there exists an adversary B with the same running time, such that $|Pr\left[b=b^{\prime }\;in\;G_{1}\right]-Pr\left[b=b^{\prime }\;in\;G_{0}\right]|\leq AdvLWE\left(B\right)$, since the circular security and LWE assumption, to distinguish **B** and **B′**, **P** and **P′** for B is almost impossible.

***G***_**2**_: In *G*_2_, the value in the generation of the challenge ciphertext **C** is substituted with uniform random elements to form matrix **C**′ in *G*_1_. The adversary distinguishes **C** and **C**′ which is as hard as solving the LWE problem, so there exists an adversary B with the same running time as that of $|Pr\left[b=b^{\prime }\;in\;G_{2}\right]Pr\left[b=b^{\prime }\;in\;G_{1}\right]|\leq AdvLWE\left(B\right)$. Notice that in *G*_2_, the values in **C** from the challenge ciphertext are independent of bit *b*, hence, $Pr\left[b=b^{\prime }\;in\;G_{2}\right]=1/2$.

In summary, AdvCPA(A) <2AdvLWE(B).

## 5. Experimental Evaluation

In this section, we conduct extensive experiments on a real network to evaluate the effectiveness of the proposed cuSCNN. We mainly focus on the following questions (RQs):

- **RQ1**: How the performance of the proposed matrix multiplication method performs;
- **RQ2**: How the proposed homomorphic matrix encryption scheme performs compared to the existing methods;
- **RQ3**: How the performance of cuSCNN on each layer compares to the state-of-the-art networks.

### 5.1. Experimental Settings

We implement cuSCNN in C++. Specifically, we use cuBLAS library to implement the matrix multiply operations on GPU, and utilize the ABT framework to implement Yao's garbled circuits. For the homomorphic matrix encryption scheme, we set the plaintext module *q* = 2^30^ (i.e., *l* = 30), which has a 30-bit length and is enough for all the intermediate values. The generation noise follows sub-Gaussian distribution with variance *var* = *q*/8*m*, *n* = 600, the security level can achieve 128.

We tested cuSCNN on two computers, both of which are equipped with Intel Xeon(R) E5-2680 CPU with 4 2.40 Hz cores, and a GeForce GTX 1080Ti GPU. The operation system is CentOS 7.9. One of them worked as client *C*, and the other play as server *S*. We took experiments in the LAN setting similar to previous work (Juvekar et al., 2018; Li et al., 2020). Each experiment was repeated 100 times and we report the mean in this paper.

The MNIST database (Modified National Institute of Standards and Technology database) is a dataset of images representing handwritten digits by more than 500 different writers. It is commonly used as a benchmark for machine learning systems. The MNIST database contains 60,000 training images and 10,000 testing images. The format of the images is 28 × 28 and the integer value of each pixel represents a level of gray with a range 0 to 255. Moreover, each image is labeled with the digit it depicts.

### 5.2. Performance of Matrix Multiplication on GPU

**Figure 5:**
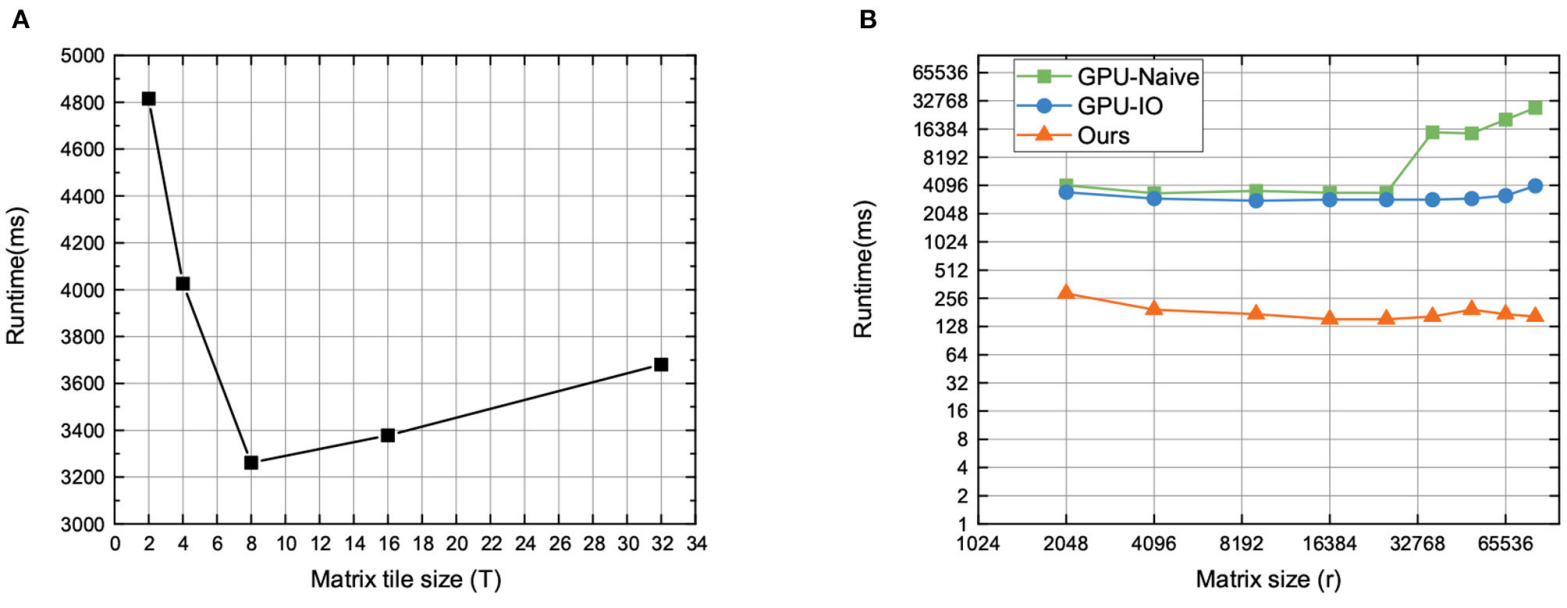
Performance of matrix multiplication methods on GPU.

In this part, we test the timing performance of proposed optimization methods on matrix multiplication, which is the core and time-consuming operation in DL-based applications. In our method, the matrix tile size (*T*) is a key factor. We set the matrix size to 1,024 (i.e., *r* = 1, 024), and the range of tile size is [2, 4, 8, 16, 24, 32]. The test results are shown in Figure 5A. We can observe that the runtime of the proposed method varies with different matrix tiles, and the optimized performance is achieved when the matrix tile size is 8. On the one hand, the inner reason is that the number of threads in each block is decreasing, but the amount of shared memory required in each block is not decreasing, after continuously increasing the matrix tile size. As a result, it will reduce the number of active threads in a streaming multiprocessor (SMP) due to the limited total number of blocks. That is, the occupancy will be reduced. In addition, calculating more elements per thread uses more registers. The number of registers in each thread will in turn affect the number of active threads in SMP, and then affect occupancy.

Then, we evaluate the proposed matrix multiplication method with two baselines on GPU. In detail, we adopt three different methods to execute matrix multiplication, including the naive way (i.e., GPU-Naive), I/O optimization (i.e., GPU-IO) method, and our optimization method. The GPU-Naive method only adopts the straightforward method to perform matrix multiplication, without considering the effect of matrix split and reunion in memory, while the GPU-IO method adopts the block matrix multiplication with matrix split, without considering the matrix reunion in memory. Figure 5B is the running time of HE.MatMult with different methods. We find that: (1) Our proposed optimization method has the best effectiveness with varying matrix size, since the running time of our methods is the lowest compared to the other methods; (2) with increasing matrix size, our method can maintain stable execution efficiency with little running time increased. That is because our method can effectively reduce the influence of IO bandwidth on performance by jointly using shared memory and registers. Furthermore, it has a higher computation efficiency via the fine-grained blocking method. Therefore, it can make more efficient use of GPU hardware computing resources.

### 5.3. Performance of Homomorphic Matrix Encryption Scheme

In this part, we test the performance of our method compared with Jiang's scheme (Jiang et al., 2018) and seIMC (Bai et al., 2020). For Jiang's method, it is a BGV-based secure matrix computation scheme that includes a novel matrix encoding method and an efficient evaluation strategy for basic matrix operations (e.g., matrix addition and multiplication). For seIMC, it is a GSW-based secure matrix computation scheme. We set the security level of seIMC and Jiang to 80 in this experiment. To achieve this security level, the cyclotomic ring dimension of our homomorphic encryption is chosen as *n* = 450, based on the estimator of Albrecht et al. (2015). The parameter settings of Jiang's and SeIMC schemes are the same as in Jiang et al. (2018) and Bai et al. (2020). Table 3 is the comparison results of the three mentioned secure matrix computation schemes. From the result, we find that: (1) Compared to the BGV-based scheme (i.e., Jiang's scheme) and SeIMC, the running time of our GSW-based scheme is faster in terms of homomorphic matrix multiplication and decryption. (2) GSW-based solutions can deal with a large-scale matrix, while Jiang's scheme fails to cope with it. Hence, the results demonstrate that our secure matrix computation solution is more suitable for real applications with large-scale data.

**Table 3:** The comparison result of homomorphic matrix encryption schemes.

**Matrix size**	**Method**	**Enc(s)**	**HE.MatAdd(s)**	**HE.MatMult(s)**	**Dec. (s)**
32 × 32	seIMC	6.998	7.345	10.639	0.0768
	Jiang's	0.09	0.01	15.592	0.0543
	Ours	0.679	0.204	**0.946**	0.067
64 × 64	seIMC	7.82	8.21	12.287	0.312
	Jiang's	0.196	0.01	37.793	0.705
	Ours	0.8	0.233	**1.24**	0.222
128 × 128	seIMC	9.843	10.402	15.824	1.305
	Jiang's	–	–	–	–
	Ours	1.127	0.291	**1.525**	0.862

*Bold values indicate our methods have a lower running time than the comparison methods*.

### 5.4. Performance Evaluation for cuSCNN

In this part, we evaluated our cuSCNN framework in an individual layer, and compared it with state-of-the-art methods. By using the proposed homomorphic matrix encryption to secure matrix computations, Conv and FC layers are the main advantage in cuSCNN. For the implementation of cuSCNN, we replace implementations of Conv and FC layers in GAZELLE with proposed optimization methods, while we also adopt the GC to perform the ReLU operation.

Runtime of each layer required for cuSCNN are presented in Table 4. Furthermore, we set *T* = 8 for all of the matrix multiplication operations on GPU.

**Table 4:** Benchmarks of cuSCNN in Conv and FC layers.

**Layer**	**Input**	**Filter/output**	**Time (ms)**			**Time per image (ms)**
			**Setup**	**Online**	**Total**	
Conv layer	(28 × 28 × 1, 846)	(5 × 5 × 1, 5)	2696.9636	0.0074	2696.971	3.19
FC layer	(846, 846)	(100, 846)	820.523	0.077	820.6	0.97
	(101, 846)	(10, 846)	760.109	0.091	760.2	0.9

In Table 4, we present the timing result of Conv and FC layers with different input sizes. We notice that: (1) Due to adopting the GPU to accelerate the online computing part, the running time of the online part is less then 1 ms either in the Conv layer or FC layer. Hence, the dominant cost of evaluating cuSCNN is that of performing the setup part, including the memory switch between CPU and GPU, the assignment, and initialization operations. (2) Compared to the FC layer, cuSCNN spends almost 3× more time executing the Conv layer's convolutional operations.

**Figure 6:**
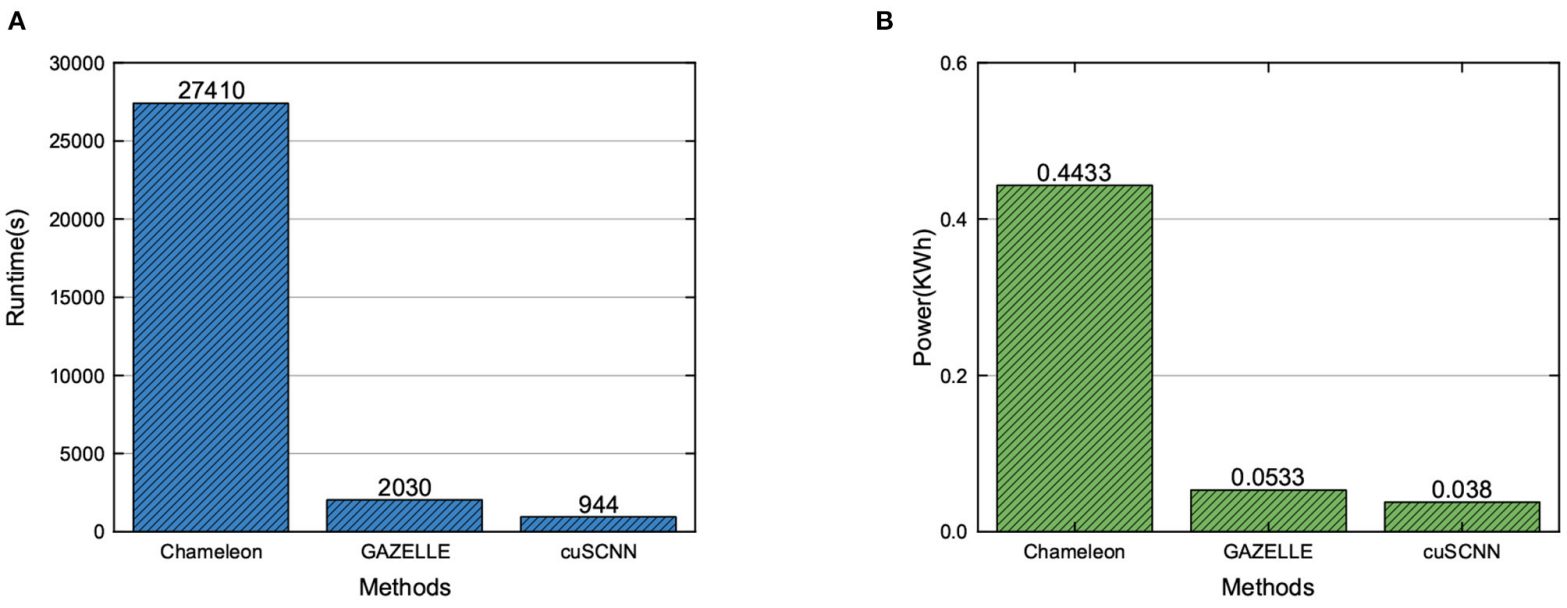
Performance comparison of privacy-preserving neural network frameworks in runtime and power consumption.

Finally, we evaluate the performance of the cuSCNN framework on the MNIST dataset, compared to the previous approaches. For comparison with previous approaches, we adopt the same CNN network architecture for all mentioned models. The CNN model takes a gray scale image with size 28 × 28 as input and has one Conv, two FC, and two ReLU layers. As the comparison framework is performed on a CPU perform, to conduct a fair comparison, we present the runtime (including computation time and communication time) and power consumptions of different models when dealing with 10,152 images. The images are able to predict with 99.1% accuracy. For the power consumption of each approach, we adopt a similar method as proposed in Tian et al. (2018). The compared results are shown in Figure 6. From the figure, we can find that: (1) Compared to the existing MPC-based framework, the mixed frameworks can enjoy a better runtime and power consumptions, which can trade-off the advantage of different secure computation technologies, as shown in Figure 6A. (2) The performance of cuSCNN outperforms GAZELLE in terms of runtime and power consumption, as shown in Figure 6B. That is because cuSCNN adopts the matrix-matrix multiplication to perform the Conv and FC layers, while GAZELLE utilizes the matrix-vector multiplication to finish these layers. Thus, cuSCNN can execute a set of images in one iteration. With the advantage of GPU's powerful computing ability, cuSCNN designed a hybrid parallel approach to implement the homomorphic matrix computations in Conv and FC layers. Therefore, it demonstrates that cuSCNN has a higher efficiency in executing the privacy-preserving neural networks.

## 6. Conclusion

The increasing popularity of cloud-based deep learning poses a natural question about privacy protection: if massive personal data are collected for model training and prediction, will this result in a rise in disclosing sensitive information? This paper focuses on tackling the privacy-preserving deep learning problem of a client that wishes to classify private images utilizing a convolution neural network (CNN) trained by a cloud server. Our target is to build efficient protocols whereby the cloud server executes the prediction task but also allows both client and model data to remain private. We find that matrix-based computations are the core operations in the neural network prediction task. However, the existing solutions have the limitations of computational efficiency and perform in a serial mode. To track it, this study proposes cuSCNN, a secure and efficient framework to perform the privacy prediction task of a convolution neural network, which utilizes the HE and GC jointly in a batch mode. The hybrid optimization approach is proposed to accelerate the execution of secure matrix computations on GPU to deal with the large-scale dataset. Extensive experiments conducted on the real network show that cuSCNN achieves a better performance on running time and power consumption than the state-of-the-art methods, when dealing with the larger-scale dataset. In the next step, we will conduct comprehensive experiments on different GPUs to evaluate the performance of the proposed method, including at the server level, desktop level, and embedded levels.

## Data Availability Statement

The original contributions presented in the study are included in the article/supplementary material, further inquiries can be directed to the corresponding author/s.

## Author Contributions

YB completed the framework's design, implemented the encryption scheme, and wrote and revised this paper. QL performed the optimization method on GPU for matrix multiplication. WW gave the main idea of the experiment flow design. YF made constructive suggestions on the organization, writing, and revision of the paper. All authors contributed to the article and approved the submitted version.

## Funding

This work was supported in parts by the National Key Research and Development Project (2020YFA0712303), in parts by Chongqing Research Program (cstc2019yszx-jcyjX0003, cstc2020yszx-jcyjX0005, cstc2021yszx-jcyjX0004), in parts by Guizhou Science and Technology Program ([2020]4Y056) and NSFC (11771421), in parts by Youth Innovation Promotion Association of CAS (2018419), in parts by the Key Cooperation Project of Chongqing Municipal Education Commission (HZ2021017, HZ2021008), in parts by CAS “Light of West China” Program.

## Conflict of Interest

The authors declare that the research was conducted in the absence of any commercial or financial relationships that could be construed as a potential conflict of interest.

## Publisher's Note

All claims expressed in this article are solely those of the authors and do not necessarily represent those of their affiliated organizations, or those of the publisher, the editors and the reviewers. Any product that may be evaluated in this article, or claim that may be made by its manufacturer, is not guaranteed or endorsed by the publisher.

## Footnotes

^1^ https://ai.google/
